# Editorial: The why of RNA granules: Form, function, and regulation

**DOI:** 10.3389/fmolb.2022.1111463

**Published:** 2022-12-13

**Authors:** Tomohiro Yamazaki, Timothy E. Audas, Natalie G. Farny

**Affiliations:** ^1^ Graduate School of Frontier Biosciences, Osaka University, Suita, Japan; ^2^ Department of Molecular Biology and Biochemistry, Simon Fraser University, Burnaby, BC, Canada; ^3^ Centre for Cell Biology, Development and Disease, Simon Fraser University, Burnaby, BC, Canada; ^4^ Department of Biology and Biotechnology, Worcester Polytechnic Institute, Worcester, MA, United States

**Keywords:** RNA granules, biomolecular condensate, micellization, nuclear condensate, membraneless organelles (MLOs), liquid-liquid phase separation (LLPS), amyloid aggregation, stress granules

RNA granules represent a broad variety of RNA and protein condensates. These granules can be constitutive or stress-induced, nuclear or cytoplasmic, static or dynamic. RNA granules have been functionally associated with a myriad of biological processes and disease states, and the depth of their physiological importance is ever growing. The aim of this Research Topic was to bring together the latest information about the form and function of a diversity of RNA granules. It was our pleasure as guest associate editors to collect and juxtapose these articles, which reveal the breadth of RNA granules and the exciting research frontiers yet to come.

A mini-review by Rhine et al. describes the dynamics of RNA granules, particularly stress granules (SGs), in the development of age-related disease. The authors frame RNA granule formation as an “inherently risky maneuver for cells”, given the propensity for these granules to transition from functional liquid-like bodies to pathogenic gel-like or solid-like aggregates in the context of aging and neurodegeneration. The article highlights the current state of imaging, sequencing, and biochemical technologies for studying RNA granules. The application of these technologies to understand liquid-to-solid granule transitions will be key to understanding and treating the formation of pathological aggregates that cause neurodegeneration.

Also related to SGs, a Perspective article by Cabral et al. collates the existing knowledge on so-called canonical and non-canonical SG subtypes. The authors focus a discussion on an oft-cited but little-studied non-canonical SG subtype, ultraviolet radiation (UV). The authors present some preliminary new evidence that suggests UV SGs may not contain mRNA and may be cell-type specific, in that cell types that experience UV regularly, such as keratinocytes, may be resistant to UV-induced SGs. The authors also highlight evidence that UV SGs, unlike most other SGs known, appear to be specific to the G1 phase of the cell cycle. They conclude by proposing that understanding the mechanisms of resistance to UV SG formation could lead to insights about how to control undesirable SG formation in the context of disease.

A review by Lacroix and Audas assessed the conservation of a variety of nuclear RNA granules from species across the eukaryotic domain. Here, the authors noted considerable diversity in the emergence and retention of these structures, as some biomolecular condensates were seen in a large cross-section of organisms, while others possessed a more limited organismal distribution. By mapping the molecular residents, regulators, and functions of these RNA granules, the authors generated a guide for examining these structures across the evolutionary landscape. As the importance of membrane-less organelles continues to emerge in disease etiology, the identification of additional species that possess these conserved structures will be fundamental for unlocking new tools and model systems for health research.

West et al. reported the roles of zinc in the formation of cytoplasmic TIA-1 condensates through liquid-liquid phase separation (LLPS). TIA-1 is a key protein in the formation of stress granules induced by various cellular stresses. TIA-1 could form fibrillar aggregates *in vitro*, but it is not present as fibrils in physiological intracellular conditions. This study used an *in vitro* phase separation assay and NMR and showed that zinc ions induce LLPS of TIA-1 together with nucleic acids *in vitro* through the interaction between RRM2 of TIA1 and zinc. This interaction also suppresses the fibrillar aggregate formation. Thus, this study provides important insights into the mechanism of how intracellular condensates are maintained as liquids without forming toxic aggregates.

In many phase separation studies, the mechanism is explained by liquid-liquid phase separation (LLPS), a type of phase separation. The following two articles deal with a new mechanism of intracellular biomolecular condensate formation called micellization, distinct from LLPS.

Yamamoto et al. reported a detailed theoretical model for the formation of nuclear bodies called paraspeckles. In this model, paraspeckles are treated as amphipathic block copolymer micelles. This micellization mechanism constructs biomolecular condensates (BMCs) with optimal size, shape, and internal structure, which are different from typical disordered spherical LLPS droplets. This model can explain recent and previous experimental observations (e.g., Souquere et al., 2010; Yamazaki et al., 2018; Yamazaki et al., 2021). Thus, this experimentally validated block copolymer micelle model of RNA-protein complexes (RNPs) would contribute to future studies on the paraspeckle and other biological block copolymer micelles not yet identified.

Yamazaki et al. reviewed the roles of RNAs scaffolding BMCs and their recent finding that BMCs use micellization of block copolymers as the formation mechanism. This review also discusses the importance of this micellization mechanism in biological contexts and designer condensates with RNA scaffolds formed by micellization. Thus, this review provides an outlook for future studies on this newly uncovered mode of action of RNPs.

Creating this article Research Topic has highlighted for us new considerations about relationships between RNA granule types and the mechanisms of their formation. RNA-mediated LLPS has recently dominated the intellectual landscape of RNA granule formation; however this Research Topic reveals that other factors, including physiological conditions, micellization, amyloidogenesis, and RNA-independent interactions also have important roles to play.
